# Induced pluripotent stem cell reprogramming: methodological evolution and challenges in clinical translation

**DOI:** 10.3389/fcell.2026.1931336

**Published:** 2026-08-21

**Authors:** Mengmeng Chen, Ning Zuo, Qi Wang, Hongyang Zhao, Pengdan Dai, Shaoshuai Liang, Wei Zhu, Haoyun Zhang, Muhammad Omer Iqbal, Dingcai Dong, Bingqiang Zhang

**Affiliations:** 1 Qingdao Engineering Research Center for Cellular Immunity and Early Cancer Screening,Qingdao Restore Biotechnology Co., Ltd., Qingdao, Shandong, China; 2 Life Valley (Qingdao) Health Technology Co., Ltd., Qingdao, Shandong, China; 3 Department of Pharmacology, School of Pharmacy, Qingdao University, Qingdao, China; 4 School of Basic Medical Science, Shandong Second Medical University, Weifang, China; 5 Neurologic Disorders and Regenerative Repair Lab of Shandong Higher Education, Shandong Second Medical University, Weifang, China; 6 College of Biological Engineering, Qingdao University of Science and Technology, Qingdao, China

**Keywords:** clinical translation, epigenetics, induced pluripotent stem cells, non-integrative vectors, regenerative medicine, reprogramming

## Abstract

Cell reprogramming can transform somatic cells into induced pluripotent stem cells providing a platform for patient-specific disease modeling, drug screening and regenerative medicine research. Since the advent of OKSM-mediated reprogramming, the system of technical approaches has evolved continuously - from integrated viral vectors to non-integrated episomal systems and, more recently, chemical reprogramming and CRISPR approaches. The simultaneous advances in single-cell multi-omics, biomaterials engineering, and artificial intelligence have further refined the controllability and precision of the reprogramming process. Despite these innovations, problems persist that hinder clinical translation: incomplete epigenetic resetting, ongoing clonal heterogeneity, genomic instability in long-term culture, and the lack of standardized Good Manufacturing Practice protocols for large-scale manufacturing. This review summarizes the trajectory of iPSC reprogramming technologies, with special emphasis on the translational applicability of each modality. We evaluated viral and nonviral delivery systems, chemical reprogramming, strategies that aid gene editing, and emerging engineering platforms, including microfluidics, smart biomaterials, and artificial-intelligence-driven process optimization. We further identify the core “translational triltrilas”, namely, the inherent tradeoffs between security, homogeneity, and scalability, and propose a comprehensive strategy to overcome these bottlenecks. By linking basic mechanistic understandings with industrial and regulatory considerations, this review aims to provide a route for transitioning iPSC technology from a laboratory tool to a clinically viable manufacturing platform.

## 1 Introduction

Reprogramming somatic cells into induced pluripotent stem cells (iPSCs) has revolutionized the landscape of regenerative medicine by providing patients with an ethically uncontroversial source of pluripotent cells. Since the discovery of OKSM, four transcription factors that reset somatic identity, the reprogramming toolbox has expanded to include viral and nonviral gene delivery, chemical compounds, and other adjunctive strategies (Takahashi and Yamanaka, 2006; Takahashi et al., 2007; Beltran et al., 2020; Guan et al., 2022). However, despite the remarkable acceleration in reprogramming technologies and mechanistic understanding, the clinical translation of iPSCs has lagged considerably behind.

This lag reflects a collection of interrelated challenges. The safety issues of incomplete clearance and residue are still unresolved; Functional heterogeneity among iPSC clones, resulting from donorspecific epigenetic memory and random reprogramming trajectories, undermines the reproducibility of therapeutic applications (Hu, 2020; Choudhury et al., 2021). In addition, the shift from a laboratory scale culture to GMP-compliant, cost-effective manufacturing poses engineering challenges that are only now beginning to be addressed (Izadi et al., 2025).

In this review, we provide a detailed summary of the evolutionary trajectory of iPSC reprogramming technologies, with a consistent focus on their capacity for clinical translation. The analysis revolves around the fundamental contradiction between safety, homogeneity, and scalability, which we refer to as the “translational triltrilas”. We investigated how different models of reprogramming might address these issues and integrated emerging engineering approaches and data-driven strategies to overcome the barriers between laboratory findings and clinical application. Through this systematic review, we aim to establish a theoretical framework to provide theoretical support for mechanistic research and translational medicine development.

## 2 Stem cell lineages and reprogramming technologies

### 2.1 Stem cell lineages and the position of iPSCs

Stem cells are defined as having the capacity for self-renewal and multilineage differentiation and are essential for development, tissue homeostasis, and repair (Li X. et al., 2022). They are generally grouped into three groups based on their origin and differentiation potential.

Embryonic stem cells (ESCs), derived from the inner cell mass of the blastocyst, are pluripotent and can give rise to all germ layer cells. However, ethical controversies over embryo destruction and the risk of allogeneic immune rejection have limited their clinical use (Liu et al., 2020).

Adult stem cells (ASCs) are present in postnatal tissues such as bone marrow and adipose tissue to maintain tissue homeostasis and mediate repair, but their differentiation capacity is lineage-limited, and their proliferative potential decreases with donor age and culture expansion (Wang et al., 2024b). It leads to morphological changes and increased secretion of pro-inflammatory factors, weakens immune regulatory function, and changes in energy metabolism state of cells, which limits the clinical application of stem cells (Rigaud et al., 2020).

**FIGURE 1:**
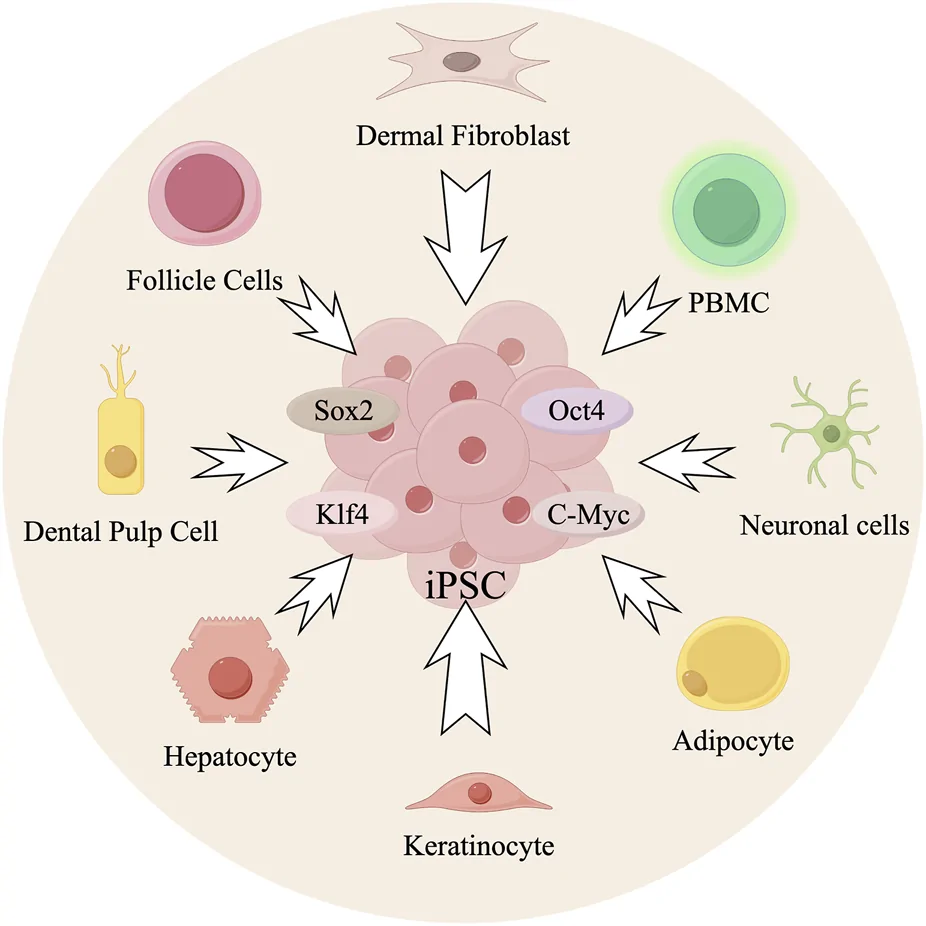
Schematic representation of somatic reprogramming. Somatic cells from a variety of different sources, such as dermal fibroblasts, follicle cells, dental pulp cells, and nerve cells, are reprogrammed into induced pluripotent stem cells through the core pluripotency network. This process highlights the plasticity of differentiated cells and the future potential of iPSCs.

iPSCs occupy a unique niche, which combines the differentiation potential of ESCs with the ethical acceptance and patient specificity of autologous ASCs by reprogramming terminally differentiated somatic cells into a pluripotent state. Making it a new central medical platform (Wang J. et al., 2024). At present, a large number of studies have reported the successful generation of iPSCs from a variety of easily obtained somatic cells, such as peripheral blood mononuclear cells and urine exfoliated cells (Figure 1), which has greatly expanded the application range of this technology (Asakura, 2014). However, it must be noted that there are still important differences between ESCs and iPSCs. ESCs are maintained in a more primitive “naive” state, corresponding to the inner cell mass before blastocyst implantation. However, iPSCs resemble the “primed” state of the ectodermal cell mass after implantation, with different transcriptional and metabolic profiles (Osnato et al., 2021; Zhou et al., 2023). In addition, iPSCs have an active glycolytic metabolism and partially retain their donor epigenetic state. This is both a patient-specific disease modeling advantage and a carefully considered variable for scale-up manufacturing (Harvey et al., 2018).

Table 1 compares the key parameters of ESCs, ASCs, and iPSCs, including pluripotency, ethical status, immunocompatibility, expansion capacity, and major technical challenges. However, these characteristics are not absolute. It is foreseeable that the risk of iPSCs will be significantly reduced as non-integrated reprogramming technologies mature.

**TABLE 1:** Comparison of characteristics of the three major stem cell types.

Characteristics	ESCs	ASCs	iPSCs
Pluripotency	Ability to differentiate into cells of the three germ layers	Limited differentiation capacity	Similar to embryonic stem cells
Ethical issues	High (involving embryo destruction)	Low	Low (generated by somatic cells)
Risk of immune rejection	High (immunosuppression required for allogeneic transplantation)	Variable (depending on the source)	Low (autograft without rejection)
Unlimited expansion capacity	Infinite amplification	Limited	Almost infinite
Key technical challenges	Ethical restrictions, immune rejection	Limited obtainable quantity, narrow differentiation potential	Epigenetic memory and potential tumorigenic risk

**FIGURE 2:**
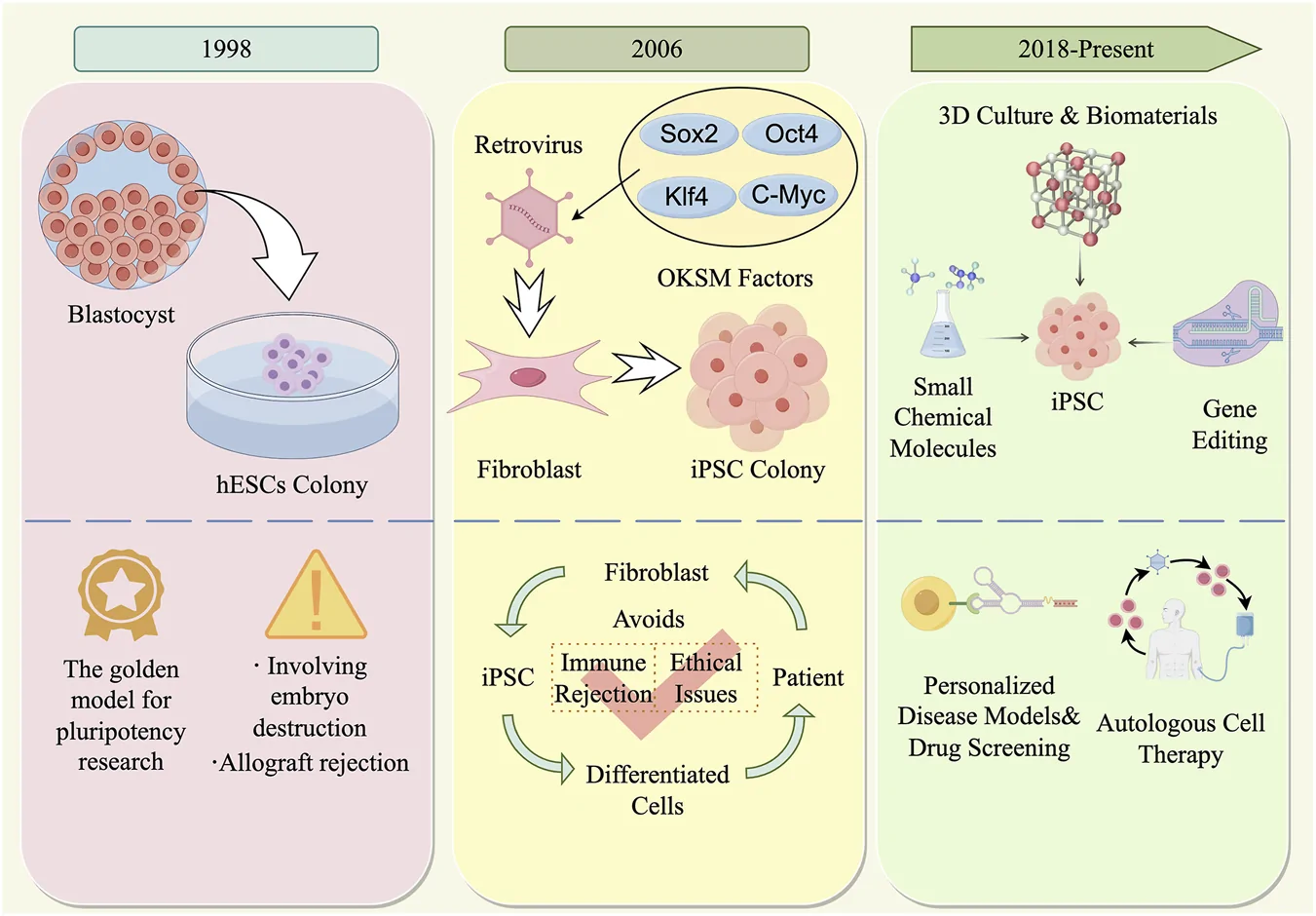
Three disruptive breakthroughs of stem cell technology. In 1998, hESCs from blastocysts became the gold standard for pluripotency with ethical concerns and rejection by allografts. In 2006, the OKSM factors enabled somatic fibroblasts to be modified into iPSCs, overcoming ethical issues and immune rejection. From 2018 to the present, the integration of 3D culture, biomaterials, small molecules, and gene editing has enabled iPSC to make progress in personalized disease modeling, drug screening, and autologous cell therapy.

The development of stem cell technology can be understood as three successive stages. The first phase (1998) established a baseline of human ESCs and provided a gold standard platform for pluripotency research, while facing ethical and immunological barriers (Thomson et al., 1998). The creation of iPSC technology in the second stage (2006) overcame the ethical constraints of ESCs, but introduced challenges related to genome integration and safety (Takahashi and Yamanaka, 2006). The third phase (approximately 2018 to present) was characterized by the pursuit of precision and safety, with the advent of chemical reprogramming, CRISPR-assisted strategies, and engineering platforms aimed at transforming iPSC preparation from a laboratory tool to a clinically viable manufacturing process (Guan et al., 2022; Li Z. et al., 2022; Zhou et al., 2024). In the future, the integration of iPSCs with cross-field and cross-technology will establish research models based on individual patient characteristics and provide a stable platform for mechanism exploration and drug screening (Figure 2).

### 2.2 Long-term evolution of reprogramming methodology

Based on the in-depth understanding of the molecular mechanism of cell fate switching, the development of reprogramming technology has been uninterrupted for 20 years, and its core driving force is the increasing demand for clinical translational and regenerative medicine platforms. The evolution of reprogramming technologies has been characterized by two parallel transitions: from integrative to non integrative gene delivery, and from empirically optimized protocols to mechanism informed, engineering enabled manufacturing systems.

The core goal of early reprogramming is feasibility, which confirms that exogenous transcription factors can forcibly reverse the intrinsic cellular properties of somatic cells, and subsequent technologies focus on eliminating the safety risks caused by viral integration (Takahashi and Yamanaka, 2006; Revilla et al., 2016). The latest research results not only pursue genetic safety, but also take into account epigenetic fidelity, experimental process controllability, and large-scale production capacity. The iteration of various technical means is an obvious continuous development vein: each new generation of technology makes up for the shortcomings of the previous generation of technology, but also generates new trade-off problems in clinical translation.

#### 2.2.1 Integrating viral vectors: basic proof of concept for reprogramming

When Yamanaka first induced iPSCs, retroviral transfection technology was used to introduce OCT4, SOX2, KLF4 and c-MYC into mouse fibroblasts (Takahashi and Yamanaka, 2006). It was subsequently applied to human somatic cells with the help of lentiviral vectors (Takahashi et al., 2007), establishing the core principle that specific transcription factors are sufficient to reshape the cellular identity of somatic cells. From the perspective of mechanism, viral long terminal repeats can efficiently mediate vector integration into the genome, drive the transcription system of host cells to continuously express reprogramming factors, form a stable signaling environment, and activate the endogenous pluripotency regulatory network (Yagi et al., 2024).

However, this mechanism also becomes a fundamental limitation of the integration approach. Integration of viral vectors into the host genome poses a two-fold risk. One is the risk of insertional mutations that may destroy a tumor suppressor gene or activate a proto-oncogene. Second, the duration of transgene expression is unpredictable, which can interfere with normal cell differentiation or induce teratoma formation (Han et al., 2017). In most of the early studies, reprogramming efficiency was consistently at extremely low levels, in part due to the stochastic nature of integration events and differential expression between transduced cells. In addition, issues related to residual epigenetic changes in somatic cells, such as DNA methylation and histone modifications, are quite common. These changes affect the subsequent lineage preference of differentiation and impair the functional integrity of differentiated cells (Hu, 2014; Singh et al., 2015).

Thus, a key consensus was reached that even successful induction of pluripotency does not automatically guarantee genome safety and epigenetic integrity and that the mode of transcription factor delivery profoundly affects the quality of the end product. It is widely accepted in the profession that integrated vectors are not clinically applicable, but their role as foundational proof of concept and benchmark for evaluating new approaches is undeniable.

#### 2.2.2 Non-integrative gene delivery systems

The inapplicability of integrated carriers in clinical applications has spurred the development of non-integrated delivery platforms. The aim is to achieve the transient expression of the reprogramming factors without causing permanent modifications to the host genome. While reducing the safety risks, it ensures sufficient bioactive factors to induce the transformation of cell fate. Currently, free-type plasmids, adenovirus vectors, and Sendai virus are widely used as mainstream non-integrating systems, each having distinct mechanism characteristics and prospects.

##### 2.2.2.1 Episomal plasmids

Episomal vectors are circular DNA molecules that can autonomously replicate outside the chromosome. They rely on viral replication initiation elements to ensure stable maintenance without genomic integration during cell division (Wang and Loh, 2019). After transient transfection, the encoded reprogramming factors are expressed for a short period of time, and as the cells continue to divide, activate PI3K/AKT relevant signaling pathways, and work synergistically to regulate endogenous pluripotency-related gene expression, thereby driving complete somatic cell reprogramming and as the cells continue to divide, the free vector is gradually diluted and eventually lost, thereby generating iPSC clones without foreign DNA (Borgohain et al., 2019; Maali et al., 2021).

Nonetheless, there are several inherent limitations to episomal vector-mediated reprogramming. The transfection efficiency of conventional plasmids in primary somatic cells is generally low, and the transient expression window may not be sufficient to overcome the epigenetic barriers that exist between different donor cell types and individuals (Chen ZhongYao et al., 2018). The overall reprogramming efficiency of episomal vectors is usually low. Moreover, the epigenetic memory carried by donor cells is difficult to completely remove, which will cause significant differences in differentiation ability between different clones (Tan T. et al., 2024). Researchers have developed modified free vectors carrying “suicide genes” such as yeast cytosine deaminase, which can selectively eliminate cells containing foreign DNA when exposed to 5-fluorocytidine, a scheme that is promising to improve the purity of non-integrated stem cell clones (Lee et al., 2019). In clinical application, free plasmids have the advantages of simple operation and low cost. However, this system relies on transient transfection, and it is difficult to achieve batch-to-batch stability without extensive optimization of delivery conditions.

##### 2.2.2.2 Sendai virus and adenoviral vectors

Adenovirus, a double-stranded DNA virus, utilizes its early genes such as E1α to deliver the OSKM factors, thereby activating the pluripotency network and initiating reprogramming (Mendoza et al., 2023). Moreover, adenoviruses have a wide range of cell infectivities and similar infectivity for cells in the division phase, resting phase, and primary cells that are difficult to transfect. This is convenient for use in reprogramming or direct trans-differentiation studies (Haridhasapavalan et al., 2019; Kisby et al., 2021). However, because the accessory proteins are easily diluted and lost during cell division, and the expression window period of the reprogramming factors is extremely short, the reprogramming efficiency mediated by adenoviruses is approximately 0.001%. Moreover, as a viral vector, its strong innate immunogenicity may interfere with the reprogramming microenvironment; theoretically, random integration is a slight possibility (Cervera-Carrascon et al., 2019; Yu et al., 2020; Nie et al., 2024).

Sendai virus, a negative-stranded RNA virus, provides a novel solution for reprogramming. The engineered Sendai virus carrying the OKSM transcript replicates and is expressed in the cytoplasm, thereby completely avoiding entry into the nucleus and integration risk. Moreover, the temperature-sensitive mutant Sendai virus can rapidly eliminate the viral particles within the cells when the culture temperature reaches 38 °C–39 °C, generating virus-free iPSC clones (Nishimura et al., 2017). The Sendai virus strategy demonstrates a higher reprogramming efficiency than adenoviruses and possesses broad-spectrum infectivity for various somatic cells, including fibroblasts and peripheral blood mononuclear cells (PBMC) (Hubscher et al., 2019; Pozner et al., 2025). A key challenge of this technology is the need for complete viral removal, as any residual material may affect the cell state. The production process needs strict contamination control to prevent viral regeneration. Additionally, the generation efficiency of PBMC and disease-related specialized cell types remains relatively low.

Both viruses can safely induce iPSCs, but the Sendai virus is more widely used in practice due to its higher reprogramming efficiency, broader applicability across cell types, and more straightforward protocol (Beltran et al., 2020). Future research in this area will focus more on improving the iteration and efficiency of viral-clearance technologies to comprehensively improve the quality and uniformity of reprogramming (Bui et al., 2019).

#### 2.2.3 DNA-free strategies: mRNA, protein transduction, and nanotechnology

The ultimate solution to genomic security lies in the complete elimination of the introduction of exogenous nucleic acids. There are two main strategies: delivery of a synthetic mRNA encoding a reprogramming factor and direct protein transduction. More recently, nanotechnology platforms have emerged as supporting tools for both delivery modalities.

##### 2.2.3.1 Synthetic mRNA delivery

Once internalized, synthetic mRNA encoding OSKM factors is translated by the host cell’s ribosomal machinery and subsequently degraded, ensuring only transient expression. Importantly, this process occurs entirely in the cytoplasm, without nuclear entry or any interaction with the host genome (Wang, 2021). This method avoids the risk of insertion mutation and residual vector DNA and is one of the safest strategies for operations relying on genetic material. Chemical modification of mRNA, including incorporation of modified nucleosides such as 5-methylcytidine and pseudouridine, as well as optimization of the 5'-end cap structure and polyadenosine tail, greatly reduces immunogenicity while improving stability and translation efficiency (Selvitella et al., 2024). The lipid nanoparticle delivery system further improved the transfection efficiency. Rajasingh et al. demonstrated that the reprogramming efficiency of human urethral epithelial cells treated with optimized mRNA-lipid nanoparticle formulations was increased by approximately 30% (Rajasingh et al., 2021).

Despite the remarkable progress in related technologies, there are still many obstacles to the large-scale clinical application of mRNA-based reprogramming. It is expensive to produce high-quality modified mRNA in large quantities. At the same time, the short half-life of messenger RNA requires multiple transfections, which leads to high complexity and cost. In non-adherent cells such as peripheral blood mononuclear cells, the reprogramming efficiency is always low, and the epigenetic memory of donor cells is often unable to be completely removed, which eventually leads to uneven quality of clonal cells (Sokka et al., 2022; Moratilla et al., 2024). Self-replicating mRNA prolongs protein expression, thereby reducing the number of transfection events required and improving overall reprogramming efficiency. However, under the GMP standard, the large-scale production of mRNA, especially the construction of fully closed automated production lines, is still a problem to be solved urgently (Peng et al., 2023).

##### 2.2.3.2 Protein transduction domains

Protein transduction domains (PTD) are short peptide sequences that directly allow macromolecules to penetrate the cell membrane. This technology fundamentally avoids exogenous integration risk by directly delivering reprogrammed transcription factors into cells as proteins using a physical method without any genetic manipulation. Harreither et al. successfully produced functional recombinant proteins and induced iPSCs by fusing PTD with factors such as OCT4 and SOX2 (Harreither et al., 2014). Son et al. fused LIN28 to a PTD derived from an insect protein (30Kc19), resulting in a fusion protein that significantly improved the reprogramming efficiency of human fibroblasts with efficient intracellular delivery (Son et al., 2022).

However, PTD technology has inherent limitations, such as a short half-life *in vivo* and a lack of cell selectivity. To this end, research has focused on more advanced delivery devices and design strategies. Recently, developed programmable protein delivery devices have synchronized protein delivery and genome editing by delivering large protein tools such as Cas9 to cells with high efficiency (Kreitz et al., 2023). Using protein language models, we can rationally design short peptides such as SLiPERs, which mimic the function of intrinsically disordered regions and can programmably recruit specific protein complexes to specific chromatin regions, enabling precise epigenetic regulation of multicellular networks (Ozkan et al., 2025). These advances suggest that direct protein delivery may become a viable clinical strategy in the future, but for now, the challenges of efficiency, cost, and scalability remain substantial.

##### 2.2.3.3 Nanotechnology enabled delivery

The nanotechnology platform integrates the fields of materials science and cell engineering to provide a new solution for the delivery of biological macromolecules. Nano-electroporation technology applies a local electric field through a nanoporous membrane, and the transfection efficiency can reach more than 90%, while the cell survival rate is maintained above 85%, which is better than traditional electroporation and lipid transfection (Ren et al., 2025). Circular RNA is more resistant to nuclease degradation and has a longer intracellular half-life than linear mRNA. The combination of circular RNA and this platform can achieve long-term and stable expression of reprogramming factors with very low immunogenicity (Krishnan et al., 2025). Zhang et al. demonstrated that the delivery of circular RNA encoding OSKM factors by nano-electroporation induced the generation of induced pluripotent stem cells with a much higher efficiency than the traditional mRNA scheme, and only a few transfection operations were required (Zhang Z. et al., 2025).

Nonetheless, there are multiple obstacles to the clinical translation of nanotechnology-based approaches. Although the physical damage to the cell membrane during nanoelectroporation is reduced, it is not completely eliminated, and the long-term effects of this operation on cell viability and genomic stability remain to be further studied (Li et al., 2025). There are still technical difficulties in the large-scale preparation of high-purity circular RNAs, especially circular RNAs with a length of more than 8,000 nucleotides and no bacterial sequence contamination are difficult to mass produce (Le Berre et al., 2025; Ma et al., 2025). In addition, the existing delivery system lacks targeting specificity, and different cell populations in a sample receive different drug loads, resulting in cell heterogeneity (Wang W. et al., 2025). Future research directions include in-depth analysis of the interaction mechanism between vectors and cells, improvement of operational controllability by combining microfluidic systems, and development of targeted delivery strategies to maximize reprogramming efficiency while reducing off-target effects (Zhang F. et al., 2025).

#### 2.2.4 Chemical reprogramming: a safe strategy without any foreign gene introduction

The chemical reprogramming technology of human somatic cells marks a qualitative leap in iPSC development. This method does not require the introduction of exogenous transcription factors or genetic material, but uses the combination of a variety of small molecular compounds to guide cells through multi-stage orderly transformation and finally reach a pluripotent cell state (Xie et al., 2017).

##### 2.2.4.1 Development context: from concept to key breakthrough

In 2013, Hou et al. reported for the first time that somatic cells in mice could be reprogrammed to a pluripotent state with the combination of a small molecule compound named “7C″, forskolin, 2-methyl-5-hydroxytryptamine, D4476, VPA, CHIR99021, E−616452, and an anti-aminocyclopropane compound. It was confirmed that cell fate transformation did not necessarily depend on exogenous transcription factors (Hou et al., 2013). However, it is much more difficult to apply this technical scheme to human cells. The core reason is that the stronger epigenetic stability of the human genome will form a higher barrier threshold for chromatin remodeling and pluripotency gene activation (Lin et al., 2022). In a landmark breakthrough in 2022, Guan et al. established a robust and reliable protocol for chemical reprogramming of human fibroblasts. This method adopts a step-by-step intervention strategy to simulate the natural regeneration pathway. In the first step, CHIR99021, 616,452, TTNPB, Y27632 and other small molecules induce epithelial-mesenchymal transition and initiate cell fate destabilization. In the second step, epigenetic modulators such as 5-azacytidine and JNK inhibitors remove DNA methylation and histone repression marks to activate dedifferentiation-related genes such as SALL4 and LIN28 A. Thirdly, chromatin remodeling compounds deregulated the OCT4 locus and activated the expression of endogenous pluripotency genes. Finally, signaling pathway modulators consolidate cellular pluripotency regulatory networks (Guan et al., 2022). The human chemoinduced pluripotent stem cells induced by this protocol can stably express OCT4, SOX2 and NANOG, and their gene expression profile and differentiation potential are similar to those of embryonic stem cells.

Subsequent modifications further accelerated and simplified the process. Wang et al. found that histone acetyltransferase KAT6A is a key epigenetic barrier during chemical reprogramming. Targeted inhibition of KAT6A by specific inhibitors can shorten the induction cycle from 50 days to less than 30 days, and greatly improve the induction efficiency (Wang Y. et al., 2025). The latest breakthrough achieved in 2025 is the transformation of human peripheral blood mononuclear cells into induced pluripotent stem cells by chemical reprogramming technology, which opens the way for large-scale production based on readily available blood bank resources (Peng et al., 2025). This development is of great clinical interest, because peripheral blood mononuclear cells are one of the most readily available sources of somatic cells for clinical use, requiring only a simple venous sampling and minimal expansion *in vitro* before reprogramming.

##### 2.2.4.2 Key mechanisms of action

The molecular mechanisms of action of chemical reprogramming differ significantly from transcription factor-mediated reprogramming schemes. Single-cell omics analysis has confirmed that small-molecule compounds can activate gene modules related to limb regeneration in vertebrates such as axolotls, induce an intermediate cell state similar to “dedifferentiation”, and make the cells transiently restore their regenerative potential (Wang G. et al., 2023). Compared with transcription factor-mediated schemes, such regeneration-like differentiation pathways can achieve more thorough removal of epigenetic modifications, which is expected to generate induced pluripotent stem cells with lower donor cell-specific memory and higher differentiation accuracy (Yang et al., 2021).

From a mechanistic perspective, small-molecule compounds induce reprogramming by targeting developmental signaling pathways such as Notch, Wnt and TGF-β. They utilize the Notch pathway to hierarchically regulate the expression of glycosyltransferases such as POGLUT1 and adhesion factors, or directly activate the transcription factors of the RXRα family, accelerating the cell reprogramming process (Jin et al., 2023; Tan Z. et al., 2024). Moreover, the small-molecule compound R406 also reduces inflammatory factor levels by inhibiting Syk and promotes mouse embryonic fibroblast reprogramming (Wang et al., 2021).

Metabolic reprogramming: The reprogramming process involves a shift in cellular energy metabolism from oxidative phosphorylation to glycolysis. Small-molecule compounds such as RG108 and Bix01294 can intervene in cellular metabolic processes, providing the necessary energy and metabolic intermediates to support fate transformation (Zeng et al., 2021).

Targeting specific barriers: Small-molecule compounds such as amicitriptamide and VPA can inhibit the clearance of aging-related genetic diseases, overcome reprogramming obstacles, reduce apoptosis or stasis during the process, maintain cell stability, and enhance reprogramming ability (Zeng et al., 2021). Wang et al. revealed that targeting key epigenetic barriers that maintain cell identity (such as the histone acetyltransferase KAT6A) can significantly promote somatic cell program silencing and pluripotency network activation, which is an important foundation for rapid reprogramming (Wang Y. et al., 2025).

**FIGURE 3:**
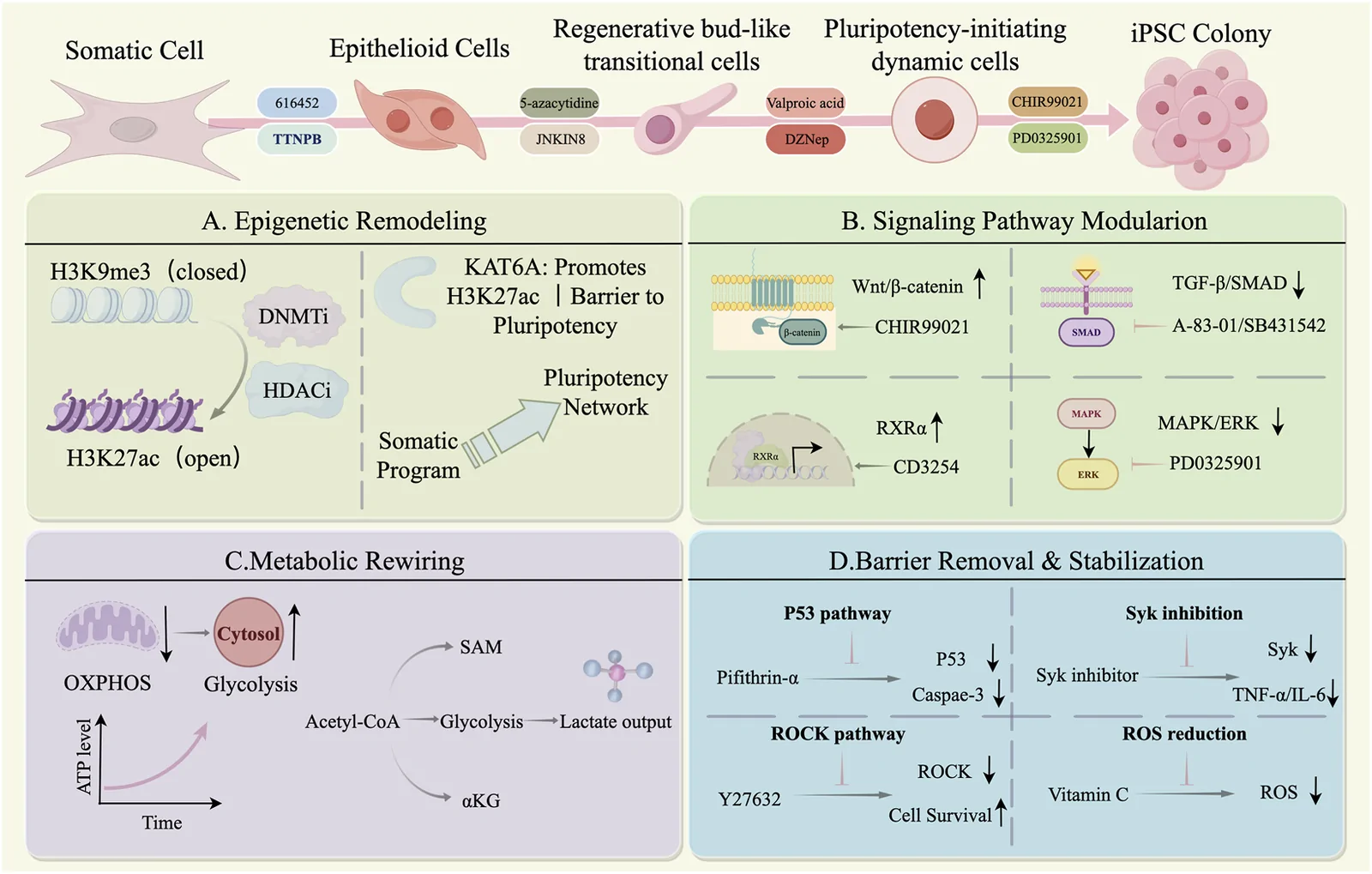
Schematic diagram illustrating the mechanisms of chemical reprogramming to induce iPSCs. **(A)** Epigenetic remodeling by inhibiting HDAC and DNMT activity on open chromatin form promotes pluripotency network construction; **(B)** Regulation of signaling pathways, including Wnt and TGFβ, removes the pluripotency barrier; **(C)** Mitochondrial metabolic reprogramming by interfering with energy metabolism provides sufficient energy support for reprogramming; **(D)** Small-molecule compounds for removing the barrier, by the action of key molecules to stabilize the regenerative intermediate state and efficiently generate hCiPSC.

These mechanisms work together to provide a multilevel, coherent regulatory framework for the induction of small-molecule compounds (Figure 3). In addition, chemical reprogramming completely avoids the core safety hazards such as vector integration, virus clearance, and exogenous nucleic acid contamination that have been difficult to solve for a long time in other programs. From the perspective of large-scale production, small molecular compounds have clear chemical composition, are easy to synthesize, and can achieve accurate quality control. Compared with biological carriers, small molecular compounds are naturally more in line with GMP production requirements.

##### 2.2.4.3 Limitations and future value

Nevertheless, chemical reprogramming still has many limitations. The efficiency of chemical reprogramming of human cells is low, and there are significant differences between different donor cell samples, especially in PBMC, which is difficult to induce, and the optimal combination of small molecules for such cells is still being studied and determined (Peng et al., 2025; Wang Y. et al., 2025). The culture cycle of chemical reprogramming is longer than that of the virus method and the additional plasmid method, which may increase the risk of genome instability and the probability of cell culture-related adaptive mutations. In addition, the exact molecular targets of various small molecule compounds in the reprogramming formulation have not been fully elucidated, which is not conducive to the analysis of their mechanism of action and directional optimization (Rehman et al., 2024).

At present, high-throughput screening technology and compound prediction biological methods based on graph neural networks are gradually making up for the above shortcomings, and can screen out compound combinations with synergistic effects, so as to clear the epigenetic memory of cells more efficiently (Chen et al., 2023; Liuyang et al., 2023). The clinical value of chemical reprogramming is not limited to the technical means themselves. Recently, pancreatic cells differentiated from induced pluripotent stem cells have been successfully transplanted into patients with type 1 diabetes, and the transplanted cells can stably colonize and normally secrete insulin, which is a key milestone for the clinical application of induced pluripotent stem cell technology (Peng et al., 2025). As the least invasive and most specific technical system in the preparation of iPSCs, chemical reprogramming is expected to become the first choice for the preparation of clinical scale iPSCs in the future if the current problems of low efficiency and poor repeatability can be solved through continuous mechanism research and process optimization.

#### 2.2.5 Engineered reprogramming factors: rational design for enhanced efficiency

Beyond optimizing delivery systems, engineering the reprogramming factors themselves has emerged as a parallel strategy to enhance reprogramming speed and efficiency. Early proof-of-concept studies demonstrated that fusing reprogramming factors with heterologous transcriptional activation domains (TADs) could markedly boost their activity (Hirai et al., 2011; Wang et al., 2011). In 2011, Wang et al. fused Oct4 and Sox2 with the VP16 TAD, achieving marked improvements in reprogramming efficiency in both mouse and human somatic cells (Wang et al., 2011). Hirai et al. further showed that the MyoD TAD fused to Oct4 could accelerate nuclear reprogramming even under serum-free conditions; notably, this enhancement was specific to the MyoD TAD, suggesting that TAD selection influences not only transcriptional potency but also the recruitment of specific chromatin remodeling machinery (Hirai et al., 2011). Zhu et al. later developed YAP TAD-fused factors (OySyNyK), increasing reprogramming efficiency approximately 100-fold compared with wild type OSNK by synergizing with TET1/2 to promote early epigenetic remodeling, establishing that engineered factors can actively reshape the epigenetic landscape rather than simply amplify transcriptional output (Zhu et al., 2014).

The field then progressed from empirical TAD fusion toward directed evolution and rational design. Veerapandian et al. established DERBY seq, a high throughput screening platform that identified artificially evolved transcription factors capable of replacing native factors (Veerapandian et al., 2018). Hu et al. further dissected the molecular determinants of improved pluripotency induction by engineered SOX17 variants (eSOX17), revealing that specific mutations at the OCT4/SOX heterodimer interface confer enhanced cooperativity (Hu et al., 2023). Most recently, Huang et al. rationally designed NanogBiD by fusing Nanog with the BRG1 interacting domain (BiD) of SS18, which specifically recruits the BAF chromatin remodeling complex to pluripotency enhancers and enables highly efficient reprogramming where wild type Nanog is ineffective (Huang et al., 2024).

Collectively, these studies reveal a conceptual trajectory: the field has moved from simply appending generic activation domains toward understanding and leveraging the specific protein interactions and chromatin regulatory complexes that govern cell fate transitions. This shift from empirical enhancement to rational, mechanism informed design represents a paradigm change that promises to make cell reprogramming not only faster and more efficient, but also fundamentally more predictable and controllable.

## 3 Quality control of reprogramming from heterogeneity to precision

iPSC reprogramming is not an instant success or failure outcome but rather a continuum of molecular states. Different states vary greatly in epigenetic fidelity, differentiation potential, and suitability for clinical applications. Even during the same reprogramming process, different monoclonal cell lines can show significant heterogeneity in gene expression, DNA methylation patterns, and differentiation potential (Hu, 2020; Choudhury et al., 2021). They arise from stochastic biological processes and donor-specific epigenetic imprinting, which poses a fundamental challenge to the clinical translation of iPSCs. Unlike small-molecule drugs and biologic agents, which can be chemically characterized and subject to strict quality control, iPSCs are living cell products whose functional properties are determined by a complex and yet incompletely resolved regulatory network.

This chapter discusses how recent advances in single-cell multi-omics, CRISPR-based epigenome editing, and epigenetic intervention strategies can improve our ability to characterize, predict, and actively regulate the mass of reprogrammed cells. Cell heterogeneity should not be regarded as an unavoidable interference factor, and precise monitoring and targeted intervention can turn this problem into an opportunity to achieve rational process optimization.

### 3.1 Single-cell multiomics: resolving the molecular map of heterogeneity

Conventional reprogramming cell population batch assays average the signals from thousands of cells, obscuring both rare subsets that successfully acquire pluripotency and transient intermediate cells that may determine the ultimate fate of the cell. The emergence of single-cell technology has fundamentally changed this research idea, which can simultaneously analyze the transcriptome, epigenome and chromatin open state at single-cell resolution (Guo et al., 2019; Schiebinger et al., 2019; Huyghe et al., 2022). With the help of such techniques, researchers have found that cell reprogramming is not a linear process, but a branching differentiation process: cells will go through a variety of intermediate states, and only a few cells will eventually differentiate into pluripotent cells (Zhu and Nie, 2025; Zikmund et al., 2025).

Single-cell RNA sequencing has shown that epigenetic imprinting inherent to somatic cells is an important barrier to inefficient reprogramming. Davis et al. used single-cell technology to screen for ubiquitin-specific peptidase 22 (USP22) and create a deletion model. It was confirmed that USP22 significantly enhanced the efficiency of iPSCs through an H2B-independent deubiquitination pathway (Davis et al., 2025). Garg et al. demonstrated that OCT4/KLF4/Sox2-regulated cells are retained in an extraembryonic endoderm state characterized by persistent expression of lineage-specific transcription factors that antagonized the pluripotency regulatory program (Garg et al., 2025). These results indicate that the efficiency of cell reprogramming cannot be improved by activation of the pluripotency regulatory network alone, but also by active inhibition of antagonistic lineage differentiation programs.

Quantitative analysis of the dosage effect of transcription factors further revealed the relevant mechanism of action. Using single-cell transcription factor sequencing technology, Liu et al. confirmed that the activation threshold of OCT4 was significantly different among different single cells, and only cells with OCT4 expression above a specific level could successfully initiate the pluripotency program (Liu W. et al., 2025). This study suggests that a strategy of using technical means to ensure uniform and adequate expression of reprogramming factors may be superior to simply increasing expression levels.

In addition to transcriptional heterogeneity, single-cell multi-omics techniques reveal a close association between energy metabolism and cell fate switching during reprogramming. The integrative analysis carried out by Fan et al. found that the metabolic transition from oxidative phosphorylation to glycolysis, accompanied by the downregulation of mitochondrial DNA copy number, is a hallmark feature of successfully reprogrammed cells (Fan et al., 2023). Rather than being an epiphenomenon of pluripotency, such metabolic remodeling is a requirement for pluripotency: pharmacologic inhibition of glycolysis blocks reprogramming, whereas increasing glycolytic flux promotes reprogramming (Hossain et al., 2023). In addition, the extracellular physical microenvironment can regulate this process through mechanical signal transduction pathways. Park et al. demonstrated that a high viscoelastic culture environment can enhance the chromatin openness of pluripotency gene loci and improve the efficiency of cell reprogramming by the synergistic effect of mechanical signals and epigenetic maps (Read et al., 2024).

Various studies using single-cell technologies have drawn a basic mechanistic picture of iPSC reprogramming, and the success of reprogramming depends on the multifaceted synergy of transcriptional programs, chromatin states, metabolic pathways, and extracellular signals. It has been confirmed that cellular heterogeneity is caused by the combination of multi-dimensional factors (Guo et al., 2019; Schiebinger et al., 2019). Such cognitive changes promote the transformation of scientific research ideas, and the design of comprehensive regulatory strategies that can overcome multiple obstacles at the same time is a future plan.

### 3.2 CRISPR-based epigenome editing: active intervention for iPSC quality control

**FIGURE 4:**
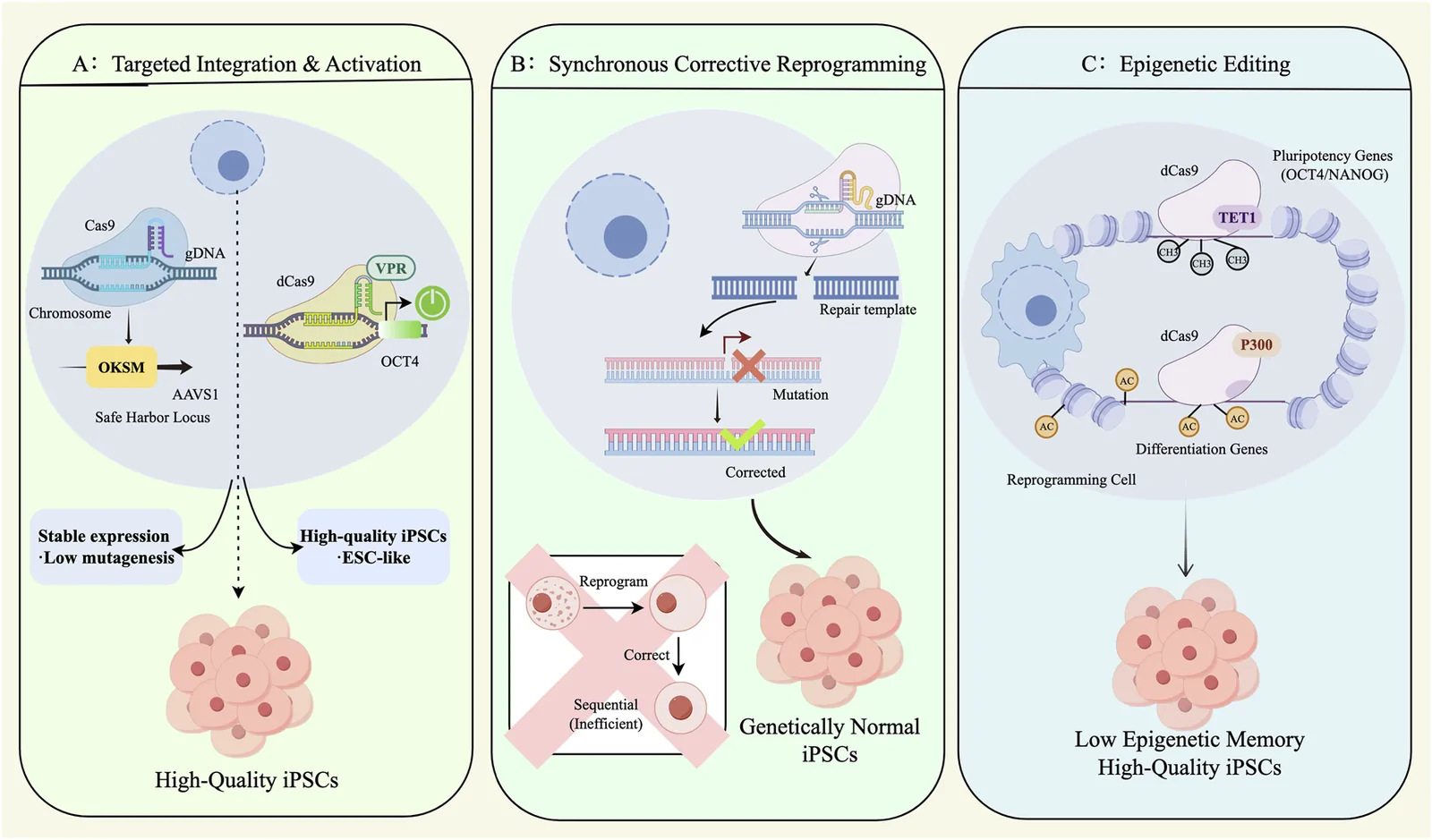
Different regulatory strategies for gene editing-assisted reprogramming. **(A)** Targeted integration and activation: OKSM factors are inserted into a safe harbor locus, enabling stable transgene expression with low mutagenesis risk to generate ESC-like high-quality iPSCs. **(B)** Synchronous corrective reprogramming: dCas9-mediated activation of OCT4 combined with mutation correction enables simultaneous gene repair and reprogramming, yielding genetically normal iPSCs. **(C)** Epigenetic editing: dCas9 fused with epigenetic modifiers (TET1, P300) targets differentiation genes to remodel their epigenetic state, reducing epigenetic memory and generating high-quality iPSCs.

As single-cell technologies identify the molecular barriers to high-quality reprogramming, CRISPR/Cas9-based editing will provide the tools to intervene in these barriers with unprecedented precision. The core of gene editing-assisted reprogramming involves directly modifying key genes or pathways that influence cell fate through editing techniques, thereby enhancing cell plasticity and reprogramming efficiency (Figure 4). The regulatory and functional aspects mainly include the following.

#### 3.2.1 Optimize the expression pattern of reprogramming factors

Gene editing achieves efficient and stable reprogramming by precisely integrating or deleting reprogramming factors at safe and targeted docking sites. On one hand, this technology utilizes CRISPR/Cas9-mediated homologous directed repair to precisely integrate reprogramming factors into safe genomic sites such as AAVS1 and CASH-1, enabling stable and controllable exogenous gene expression and markedly diminishing the likelihood of insertional mutations (Javaid and Choi, 2022). The CRISPR/Cas9 targeted integration system designed by Sung et al. regulates pathways such as Wnt/β-catenin and P53 to break the differentiation barrier of somatic cells, thereby enhancing the reprogramming efficiency and obtaining iPSCs without any residual exogenous genes (Sung et al., 2021). On the other hand, the nuclease-dead Cas9 (dCas9)with a transcriptional activation domain was used to precisely activate endogenous pluripotency genes. Chavez et al. fused dCas9 with the transcriptional activation complex VPR, specifically binding to and activating endogenous OCT4 expression, resulting in iPSCs of higher quality that were more similar to ESCs (Chavez et al., 2015).

#### 3.2.2 CRISPR-assisted synchronous correction

The combination of CRISPR gene editing technology and cell reprogramming technology adds a second guarantee to the quality control system: the repair of pathogenic gene mutations can be completed simultaneously in the process of reprogramming. For iPSCs with mitochondrial disease, CRISPR/Cas9 correction restores the function of *ATP1A2*, improves mitochondrial homeostasis, and avoids interference with iPSC function by disease manifestations (Sladen et al., 2021). Using the CRISPR/Cas9 system to repair ABCA4 mutations and induce their reprogramming into iPSCs can achieve the synchronous corrective reprogramming of Stargardt disease cells and generate genetically normal iPSCs (Siles et al., 2023). Similar patterns of synchronous corrective reprogramming have been reported in monogenic diseases such as aortic aneurysm and Fabry disease (Zhou et al., 2021; Klein et al., 2024). In contrast to the traditional “reprogramming first and then correction” step-by-step strategy, this technology can obtain genetically homogeneous cell lines more quickly, effectively enhancing disease modeling precision and drug screening reliability.

However, the synchronous operation system will introduce more experimental variables and needs to be refined for condition optimization. The efficiency of homologous directed repair in cell reprogramming is usually lower than that in actively proliferating cell lines. At the same time, the cytotoxicity caused by Cas9 protein introduction will further reduce the already low reprogramming efficiency (Wen et al., 2018). At present, some of the above problems have been alleviated by improved means such as high-fidelity Cas9 mutants and optimized guide RNA sequences, but the balance between editing efficiency and cell activity is still the core consideration for experimental scheme design.

#### 3.2.3 Epigenetic memory and its clearance

Epigenome editing tools enable targeted rewriting of epigenetic marks at specific genomic sites without altering the underlying DNA sequence. Inactivated Cas9 (dCas9) was fused to the catalytic domain of TET1 to actively demethylate CpG islands in the promoter regions of pluripotency genes, accelerating their activation during reprogramming. Similarly, dCas9-p300 fusion protein can add histone acetylation modification at the silenced gene site to improve chromatin opening and facilitate transcription factor binding (Ha et al., 2022; Moura et al., 2025). Moreover, Wang et al. confirmed that targeted demethylation of abnormal methylation sites on the promoters of OCT4 and NANOG in the early stage of reprogramming could significantly improve the preparation efficiency of induced pluripotent stem cells and improve the monoclonal homogeneity of cell lines (Wang P. et al., 2023).

The timing of epigenetic editing and reprogramming is crucial. Epigenetic modifications are not independent factors in determining cell fate, but rather regulators that regulate the binding of transcription factors to DNA regulatory elements. Recent studies have shown that the greatest improvement in monoclonal cell quality can be achieved by early intervention in the first few days of reprogramming, before cells have become pluripotent or differentiated. If the intervention time is delayed, the optimization effect will continue to weaken (Guo et al., 2025). This temporal sensitivity highlights the need to integrate epigenome editing into precise, timed delivery protocols, which are better suited to mRNA -based dCas9 delivery than vector systems, where termination of sustained expression is difficult.

#### 3.2.4 Off-target effects and genomic stability

The use of CRISPR editing in iPSC generation poses critical safety risks. High-fidelity Cas9 mutants could reduce off-target cleavage, but could not completely eliminate it. This can lead to unexpected mutations that are difficult to identify by conventional quality control methods. In addition, the DNA damage caused by Cas9 cleavage will activate the DNA damage response pathway, and then induce the activation of the p53 protein, eliminate cells with normal functions, increase the risk of tumor formation, and cause safety contradictory effects (DuBose et al., 2022; Nakano et al., 2022; Afanasyeva et al., 2023). The long-term genome stability of CRISPR-edited iPSCs remains a focus of research. Feuer et al. used the CRISPR-Del/Rei strategy to achieve scar-free accurate editing, shorten clone screening time, and reduce off-target risk (Feuer et al., 2022). Jurlina et al. applied high-fidelity Cas9 variants to reduce non-specific cleavage (Jurlina et al., 2022), Suter et al. used a pilot editing technique with optimized pegRNA and protein variants to directly integrate into the somatic reprogramming process, improving the feasibility and safety of complex editing (Suter et al., 2025).

For gene editing induced pluripotent stem cell lines for clinical application, strict quality control must be carried out, and detection methods should cover whole genome sequencing, karyotype analysis and long-term cell status monitoring. Although CRISPR-related technology is a powerful tool to optimize cell quality, before clinical application of CRISPR-related technology, a comprehensive risk-benefit assessment should be carried out, and a comprehensive safety standard system should be established to take into account both the target editing effect and various unexpected side effects caused by the editing process.

### 3.3 Toward predictive quality control

The ultimate goal of cell quality control is to predict in advance which cell clones will be able to function safely and effectively in the patient’s body before transplantation. Currently, most of the quality assessment methods for iPSCs rely on retrospective characterization tests that require a significant amount of time and experimental resources to obtain results, including karyotype analysis, expression detection of pluripotency markers, and teratoma formation experiments, etc. (Matiukhova et al., 2025). The integration and application of single-cell technology and gene editing intervention are gradually transforming the existing model and promoting the implementation of pre-informed quality prediction.

By integrating multi-omics data sets, machine learning methods have been used to identify early transcriptional and epigenetic signatures that predict the state of reprogramming. Single-cell RNA sequencing was also used to map the network of marker gene expression patterns associated with high-quality iPSCs (Jain N. et al., 2024). By applying this prediction method to the chromatin open mapping models generated at the early stage of reprogramming, it may be possible to predict the pluripotency potential of single-cell clones with high accuracy. Combined with an automated culture platform, real-time quality monitoring and closed-loop process control can be achieved.

This paradigm shift in quality control could have profound implications at the regulatory level. At present, supervision relies on the detection of several quality indicators and release standards for the final product (Matiukhova et al., 2025). With the maturity of predictive quality control technology in the future, the industry may shift to a new quality assurance model with process flow as the core. By forecasting and monitoring the key process flow, the consistency of the product batch can be more effectively ensured. However, to achieve this transformation, it is not only necessary to complete a series of comprehensive technical verification work, but also to harmonize the regulatory standards of various countries, which is a difficult but extremely meaningful task.

## 4 Engineering strategies for controlled and scalable iPSC manufacturing

**FIGURE 5:**
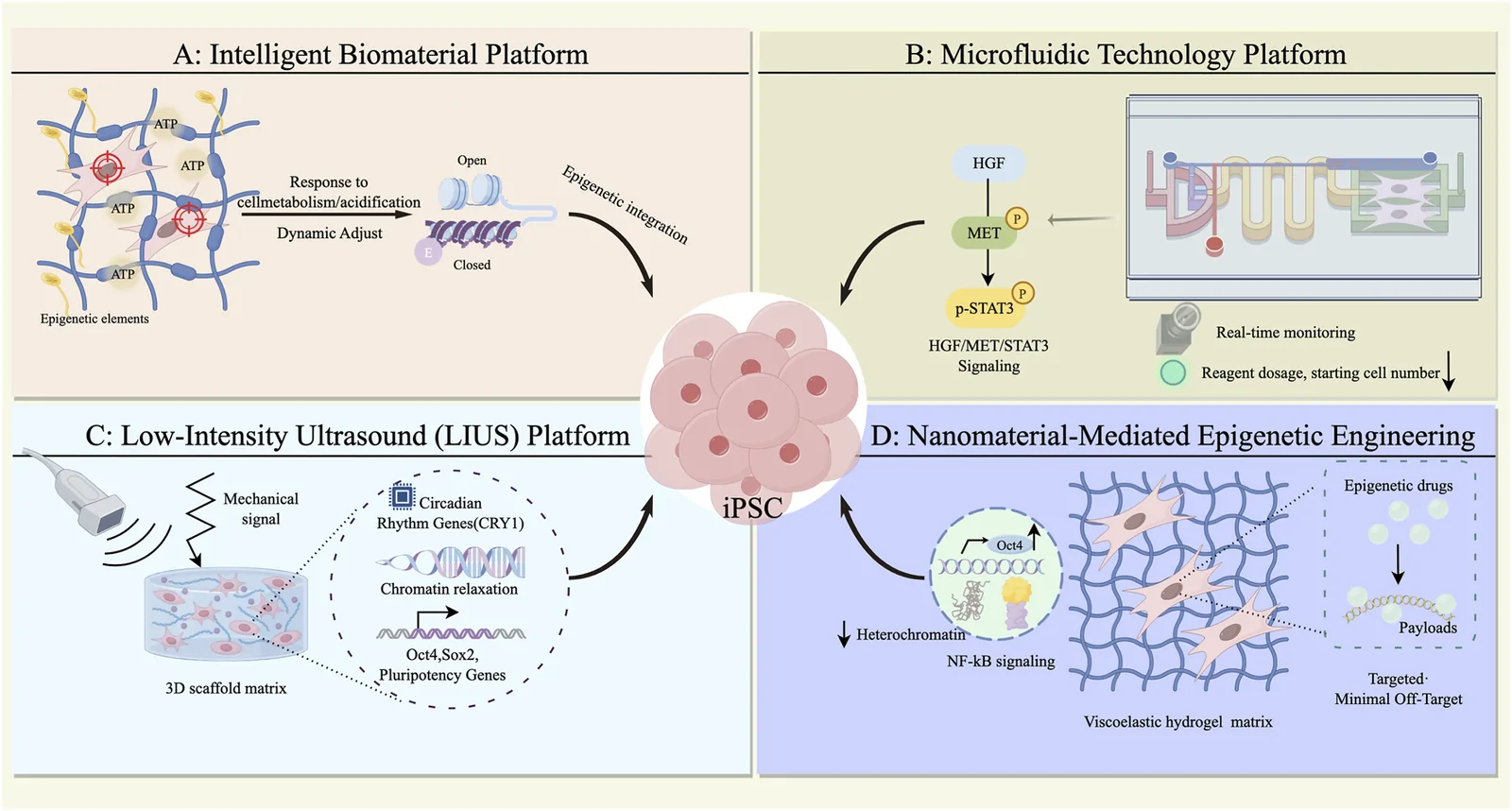
Co-engineered regulatory approaches to iPSC reprogramming. **(A)** Intelligent biomaterials platform: ATP-responsive materials sense cellular metabolism to dynamically release epigenetic elements. **(B)** Microfluidic platform: real-time monitoring of changes in the culture microenvironment, through HGF/MET/STAT3 signals to achieve precise regulation. **(C)** Low-intensity ultrasound platform: LIUS promotes the efficient generation of iPSCs by regulating a circadian gene (CRY1) to alter the chromatin open state. **(D)** Nanomaterial mediated epigenetic engineering: Delivery of epigenetic drugs in hydrogel matrices targeting heterochromatin remodeling via NF-κB signaling allows precise modification with minimal off-target effects.

In the direction of homogeneous clinical grade iPSC production, it is far from enough to rely on the optimization of reprogramming efficiency summarized above. The effect of cell reprogramming is not only determined by genetic or chemical conditions, but also profoundly influenced by the physical microenvironment in which the reprogramming process occurs. Engineering approaches must be used to address the physical, mechanical, and operational aspects of iPSC production. Through various engineering systems, including biomaterials, microfluidic technology, automation equipment and artificial intelligence (Figure 5). The combination of these technologies can achieve precise regulation, real-time monitoring and large-scale preparation, and the above capabilities are the core basis for the clinical translation of this technology. Engineering platforms that can actively construct a microenvironment, monitor cell status, and iteratively optimize process parameters are the key links between mechanism research and mass production of cell therapeutic drugs.

### 4.1 Smart biomaterials: constructing reprogramming microenvironments

The extracellular microenvironment exerts potent regulatory effects on cell-fate decisions through biochemical and biophysical signals (Read et al., 2024). Smart biomaterials such as hydrogels and artificial matrices that can be engineered to sense and respond to the state of cells can be used to construct programmable scaffolds that actively participate in the process of cell reprogramming instead of acting as inert support carriers. Its physical properties, including stiffness, viscoelasticity, and surface topology, directly regulate cellular epigenetic status and gene expression through mechanotransduction pathways that connect the extracellular environment to the nucleus (Le et al., 2019; Indana et al., 2021). Wu et al. demonstrated that a viscoelastic matrix significantly improved the efficiency of reprogramming fibroblasts into iPSCs compared with a purely elastic matrix. Mechanologically, the viscoelastic environment can downregulate the heterochromatin mark H3K9me3 level and improve chromatin accessibility at the promoter regions of pluripotency genes, thereby reducing the epigenetic barrier of cell reprogramming. This effect is mediated by the cytoskeleton-nucleus mechanical transduction pathway: matrix viscoelasticity can regulate actomyosin contractility, and at the same time regulate the transport of mechanosensitive transcriptional regulators into the nucleus (Wu et al., 2025). The above findings suggest that the physical microenvironment is not a neutral background but rather an active regulator in cell fate decisions.

In addition to static mechanical properties, advanced biomaterials can provide time-controlled dynamic signals to adapt to the changing physiological needs of cells during reprogramming. Zhu et al. constructed an ATP-responsive hydrogel that can sense cellular metabolic activity and release epigenetic regulators in proportion to the metabolic level of the encapsulated cells (Zhu et al., 2024). When cells gradually transition to a pluripotent state and ATP produced by glycolysis increases, the hydrogel will accelerate the release rate of active substances and strengthen the epigenetic intervention at the most critical transition node of cell differentiation. If controllable release elements that can be activated by specific cellular proteases or metabolites are added to the hydrogel matrix, spatiotemporal precise regulation that cannot be achieved by soluble drug addition can be achieved (Wang X. et al., 2024). The combination of such intelligent biomaterials with conventional culture protocols can greatly improve the efficiency of cell reprogramming and clonal uniformity, especially for difficult-induced cell samples.

### 4.2 Microfluidic platforms: miniaturization and precise control

Microfluidic devices can realize the precise regulation of liquid flow, material transport and chemical concentration gradients at the micrometer level, which provides a new technical route for the reconstruction of the reprogramming microenvironment. At the same time, it has multiple advantages that are difficult to achieve in traditional culture systems: lower reagent consumption, more controllable cell-cell interactions, and real-time observation of cell behavior (Panariello et al., 2023).

It has been confirmed that microfluidic technology can build a more uniform and stable controlled microenvironment culture condition, thereby improving the effect of cell reprogramming. Jain et al. developed a simplified microfluidic protocol to successfully reprogram fibroblasts into iPSCs and further induce their differentiation into neural stem cells. Compared with the standard experimental protocol, the reagent consumption was reduced by two orders of magnitude, and the starting cell dosage was reduced by nearly 90% (Jain S. et al., 2024). This drastic reduction in cell consumption is of great value for clinical research where donor cell sources are limited. From the perspective of molecular mechanism, the improvement of reprogramming efficiency under microfluidic system is closely related to the activation of HGF/MET/STAT3 signaling pathway, which can promote the survival and proliferation of reprogramming intermediate cells (Panariello et al., 2023). By precisely regulating the concentration gradient of soluble factors and fluid shear stress, the microfluidic system can simulate the *in vivo* developmental microenvironment characteristics that are missing in static culture, thereby reducing the random differences caused by traditional reprogramming.

In addition, the microfluidic platform can actively sort cells based on the expression level of pluripotency markers and the real-time detection results of cell metabolic activity. The direct physical separation of high-quality cell clones in the early stage of the experiment can not only reduce the manpower and cost required for manual selection and expansion of clones, but also is expected to realize the construction of a higher throughput and qualitative iPSC cell bank (Panariello et al., 2023; Qu et al., 2023).

### 4.3 Physical stimuli and nanomaterial-assisted interventions

In addition to smart biomaterials and microfluidics, physical stimulation and nanomaterials can serve as complementary strategies to modulate the outcome of cell reprogramming. Low-intensity ultrasound can output controllable mechanical signals to regulate chromatin structure and gene expression through mechanobiological mechanisms, providing a new tool for cell reprogramming (Wu et al., 2024). In the three-dimensional culture system, it has been confirmed that ultrasound stimulation can promote epithelial-mesenchymal transition, improve the generation efficiency of induced pluripotent stem cells by regulating histone modification, and reduce clonal heterogeneity. This effect is related to circadian gene regulation pathways such as cryptochromes 1 (CRY1) (Generoso et al., 2023; Kim et al., 2024). Although LIUS has the advantage of adjustable multiple parameters such as frequency, intensity and action duration, which can optimize the action conditions for different cell types, the lack of standardized ultrasonic treatment protocols limits its routine application in clinical preparation processes.

Nanomaterials with programmable surface chemistry and high drug loading capacity enable the targeted delivery of epigenetic regulators. Compared with the direct addition of soluble drugs, nanoparticle systems encapsulated with histone deacetylase inhibitors or DNA methyltransferase inhibitors can achieve sustained drug release, which can improve the accessibility of pluripotency genes while minimizing off-target effects (Ishii et al., 2024). Composite systems constructed by combining nanomaterials with synthetic matrices can produce synergistic effects in chromatin remodeling and regulation of accessibility of pluripotency genes (Jiang et al., 2024). However, the problems of nanoparticle preparation, such as batch differences, unclear long-term cytotoxicity, and lack of standardized procedures, are still obstacles to its clinical translation (Younis et al., 2022). Although related research is still in its infancy, low-intensity ultrasound and nanomaterials together expand the regulatory tool library of cell reprogramming microenvironment and break through the limitations of traditional regulation.

### 4.4 Automation and artificial intelligence: toward intelligent manufacturing

Various innovative iPSC generation systems have great efficacy. But the resulting combinatorial complexity will rapidly exceed the limits of manual processing optimization. Therefore, with the support of automation and artificial intelligence technology, the potential of iPSC preparation process can be fully released, a systematic and uniform process scheme can be realized, and a quality control system for prediction rather than remediation can be established.

The feasibility of high-throughput iPSC generation has been confirmed by automated platforms that can perform multiple concurrent reprogramming schemes, which can reduce manual error and improve batch-to-batch consistency. The automated system built by Tristan et al. completed about 24 reprogramming experiments within 50 days, maintained the purity of iPSCs throughout the process, and achieved a processing throughput that could hardly be achieved by manual operation (Tristan et al., 2021). At present, some automated systems have real-time imaging and non-invasive detection functions, which can screen and enrich iPS clones with developmental potential in the early stage of reprogramming (Piotrowski et al., 2021). The core of automation technology is not to simply replace human labor, but to eliminate the operational differences between operators, which is the main reason for the instability of cell preparation products. Even if the standardized experimental process is adopted, there are slight deviations in the time control, reagent ratio, cell treatment and other aspects of the operation by different technicians, and the final experimental results will be significantly different (Chen et al., 2024). However, the high acquisition and maintenance costs of robotic equipment make it difficult to widely apply the technology outside professional research centers.

Machine learning systems, especially Bayesian optimization and reinforcement learning methods, can realize the rational selection of complex parameter spaces. Dallal et al. demonstrated that Bayesian optimization could efficiently screen out the optimal formula suitable for delivery and significantly reduce the number of experiments required compared with traditional experimental design methods (Dalal et al., 2024). Similar methods have been used to optimize the concentration of key small molecule compounds such as CHIR99021 to achieve a balance between efficacy and toxicity. In addition to parameter optimization, AI systems can also predict the outcome of cell reprogramming based on early experimental data. Arnold et al. integrated multi-omics data sets into multi-dimensional association models and identified DDK3 as a previously undiscovered suppressor of cellular reprogramming; After subsequent knockout of DDK3 gene, the preparation efficiency of induced pluripotent stem cells was significantly improved (Arnold et al., 2021).

Predicting clonal cell quality on the basis of morphological features is another area of research that is growing rapidly. Through targeted training on reprogramming culture time-lapse images, deep learning models that can identify early colony morphology related to subsequent pluripotency and genomic stability can achieve non-invasive quality detection at a time point much earlier than traditional staining or sequencing techniques (Chu et al., 2023). When used with an automated culture platform, closed-loop regulation can be achieved: the system monitors cell status in real time, adjusts process parameters for detected deviations, and selectively enriches clone cells that are predicted to meet quality standards.

The application of automation technology and artificial intelligence is gradually changing the iPSC preparation from manual and empirical laboratory technology to a uniform production system. Through the mechanized automatic platform to perform the operation, avoid human interference and error; The input materials and production process parameters were detected in real time, and the best regulation scheme was predicted by artificial intelligence to stabilize the cell culture process in the range of continuous output. At the same time, the final product quality can be predicted according to the early process data, so as to realize the immediate release judgment (Nath et al., 2023).

This vision is still at the stage of conception, and there are still multiple obstacles to overcome. The integration of automation equipment platforms and artificial intelligence prediction models requires a perfect underlying data architecture and standardized data formats, and the relevant supporting systems have just started. The regulatory compliance verification of artificial intelligence-based quality prediction techniques requires a large amount of supporting data to prove that the reliability of such prediction models is at least not less than that of traditional final product inspection methods. In addition, the capital investment and professional technical reserve required for the landing of such technologies are difficult for small scientific research teams to afford (Idnay et al., 2024).

Despite these hurdles, the direction is clear: the field is moving toward entirely new manufacturing systems. In this system, the results of cell reprogramming are no longer reviewed after the fact, but rely on comprehensive biological theory, engineered regulatory methods, and data-driven predictive models to achieve prospective and directed design.

## 5 Clinical translation: progress, barriers and future directions

### 5.1 Clinical progress: from first-in-human to emerging applications

The clinical translation of iPSC technology has developed rapidly in the fields of ophthalmology, neurology and metabolic diseases. This field is characterized by easy access to target tissues, thorough disease mechanisms, and a large number of unmet clinical treatment needs. A series of early clinical studies provided key proof-of-concept data for the safety, tolerability, and potential efficacy of iPSC-derived cell products.

#### 5.1.1 Ophthalmology

The eye is an ideal target for iPSC therapy due to its immune immunity, easy drug administration and monitoring, and clear pathogenesis research. Age-related macular degeneration (AMD) is the leading cause of blindness in the elderly, and it is also the core direction of iPSC clinical research in ophthalmology. A number of early clinical trials have been carried out to evaluate the transplantation of iPSC-derived retinal pigment epithelial (RPE) cells, and relevant studies have confirmed that transplanted cells can stably colonize without serious adverse events such as tumorigenesis and ectopic tissue formation (Akiba et al., 2023). Mandai et al. performed the world’s first autologous iPSC-RPE transplantation for AMD patients, and the follow-up results of several years showed that the transplanted tissue survived and the patient’s vision remained stable (Mandai et al., 2017). Subsequently, HLA-matched or immune escape modified allogeneic iPSC-RPE cell lines will be used to further expand the application feasibility of off-the-shelf transplantation protocols. On the other hand, patient-derived iPSCs carrying gene mutations such as RHO, ABCA4, and RPGR have been differentiated into photoreceptor precursor cells or retinal pigment epithelial cells for transplantation, and preclinical experiments have confirmed that transplanted cells can achieve functional integration and improve visual function (Liu B. et al., 2025). Although such therapies are still in the early stage of clinical evaluation, iPSC technology combined with gene editing technology may be able to repair the pathogenic mutations in cells before transplantation, which is expected to achieve the radical cure of monogenic retinal diseases (Siles et al., 2023).

#### 5.1.2 Neurological disorders

Parkinson’s disease has been the focus of iPSC cell therapy research. Clinical trials have confirmed that iPSC-derived dopaminergic neuronal precursor cells can survive after transplantation, produce dopamine and improve motor function (Sawamoto et al., 2025). These early results provide encouraging evidence that cell-replacement therapies for neurodegenerative diseases are technically feasible, although efficacy is currently underevaluated. In addition, the neuron model constructed by iPSCs can complete drug screening and mechanism studies. Motor neurons derived from iPSCs from patients with sporadic ALS recapitulated key pathological features. A variety of lead compounds that can alleviate this pathological phenotype have been identified by high-throughput screening (Bye et al., 2026). Similarly, iPSC-derived microglia have revealed the role of neuroinflammation in the progression of Alzheimer’s disease and identified potential therapeutic targets that were difficult to detect through animal models (Park et al., 2023).

#### 5.1.3 Diabetes

The differentiation of iPSCs into functional pancreatic β cells is one of the most difficult goals in clinical application, which requires the construction of a complex endocrine organ with both precise blood glucose monitoring and insulin secretion functions. Du et al. demonstrated that iPSC-derived islet-like organoids could ameliorate diabetic symptoms in nonhuman primates, providing critical preclinical validation for clinical application in humans (Du et al., 2022). Subsequently, autologous iPSC-derived islet cells were transplanted into type 1 diabetic patients *in vivo*, which showed that endogenous autonomous and physiological blood glucose control was restored after transplantation, and they were completely and stably separated from insulin injection therapy 75 days after transplantation, and the effect has been stable for more than 1 year now (Wang et al., 2024a).

### 5.2 Persistent barriers to clinical translation

#### 5.2.1 Tumorigenicity and genomic instability

Residual undifferentiated iPSCs carry the risk of tumor formation, which is the most serious safety concern in clinical translation. Preclinical studies have shown that teratomas can be induced even in small numbers of undifferentiated iPSCs, and the long-term risk of malignant transformation of cell clones with genetic or epigenetic abnormalities cannot be completely eliminated (Yasuda et al., 2018). Existing strategies, including terminal differentiation protocols, sorting to remove residual pluripotent cells, and the application of suicide gene systems, can only reduce this risk, but cannot completely eradicate it (Matiukhova et al., 2025).

In addition, conventional quality-control methods cannot ensure the genomic integrity of iPSCs. Single nucleotide variation, copy number variation and chromosome aberration will accumulate during long-term cell culture. Moreover, the selective pressure generated by the reprogramming process may also enrich cell clones carrying TP53 mutations or other oncogenic mutations (Panferov et al., 2025). Although conventional karyotyping and whole-genome sequencing can detect large-scale genomic abnormalities, they cannot rule out all clinically harmful mutations. The development of comprehensive, low-cost genomic surveillance methods that can be integrated into clinical-grade cell manufacturing workflows is a central imperative.

#### 5.2.2 Epigenetic incompleteness and functional variability

The epigenetic memory of donor cells and the incomplete reset of DNA methylation and histone marks continue to affect the functional stability of iPSC-derived products (Hu, 2014; Singh et al., 2015). Even if iPSCs have good pluripotency and can differentiate into the target cell lineage, they are still affected by the residual donor-specific epigenetic patterns, which will bias the differentiation process toward the original donor cell lineage, reduce the generation efficiency of target cells, and change the functional properties of the end product (Scesa et al., 2021; Poondi Krishnan et al., 2023). The regulatory implications of epigenetic variability are striking. The current regulatory system highly relies on marker expression detection and functional identification at the time of product release, which cannot fully reflect the epigenetic status of cells and makes it difficult to predict the long-term biological behavior of cells. The development of epigenetic quality evaluation indicators that can be included in the batch release test is essential to ensure the uniformity of products between different production batches and different clinical application sites.

#### 5.2.3 Scalability and economic viability

The transition from laboratory-scale production to clinical-scale manufacturing remains one of the most significant challenges in the clinical application of iPSCs. The cost required for producing patient-specific iPSC-derived cell products is extremely high, severely hindering their large-scale clinical application (Adegunsoye et al., 2023). Although the immunogenicity of iPSCs can be eliminated through large-scale screening or gene editing technologies, and universal cell products that directly provide ready-to-use cell sources can be developed, this approach also brings new challenges: including immune compatibility management, long-term stability verification, and coordination issues regarding intellectual property and regulatory approval (Ahmad et al., 2025).

The manufacturing bottleneck not only involves cost issues but also capacity limitations. Currently, production facilities that meet GMP standards are extremely scarce; to expand the iPSC production process to meet the needs of large-scale clinical applications, a significant amount of infrastructure construction, the recruitment of professional personnel, and the optimization of the supply chain are required (Haase et al., 2025). Developing a closed and automated production platform that integrates the entire process of cell reprogramming, differentiation, purification, and formulation preparation is crucial for achieving the high output and reasonable cost structure required for commercialization.

### 5.3 Future directions: toward integrated clinical solutions

#### 5.3.1 Universal cell banks

Developing a universal iPS cell library can effectively resolve the contradiction between personalized preparation methods and the current methods. By establishing an iPS cell library on a large scale from donors representing the most common HLA haplotypes, it is theoretically possible to cover the majority of the population, which can not only reduce the demand for autologous cell preparation but also maintain immune compatibility (Ahmad et al., 2025). Based on CRISPR technology, editing the HLA I and II genes in a small number of master cell lines can cultivate universal donor cells that do not require HLA matching and can evade immune recognition (Thongsin et al., 2023). Although both of these methods face technical and regulatory challenges, they undoubtedly represent the most promising development path for achieving large-scale, low-cost iPSC therapies.

#### 5.3.2 Integrated process development

Optimizing only a single parameter in the process flow cannot meet the requirements for clinical stability. Integrated process development integrates reprogramming, differentiation, purification and formulation preparation into a unified system, which is expected to achieve a closed-loop production mode with real-time process control and predictive quality release functions (Haase et al., 2025). This technical solution requires cross-disciplinary collaboration across traditional boundaries, establishing a cooperative alliance, sharing research infrastructure and open databases, in order to promote the rapid development of technology in this field.

#### 5.3.3 Regulatory coordination and quality standards

Currently, there are numerous institutions conducting iPSC research worldwide. The regulatory systems vary significantly across different regions, with differences in requirements for product characteristic identification, preclinical safety studies, and clinical trial design. Establishing unified quality management standards for iPSC preparation, characterization, and storage will alleviate the burden of compliance regulation and facilitate the conduct of multi-center clinical trials (Madrid et al., 2024). To achieve unified and coordinated regulatory standards, joint collaborative projects involving researchers from research institutions, industry stakeholders, and regulatory agencies are indispensable.

#### 5.3.4 From individualized to population-level impact

The goal of iPSC technology is to provide safe, effective and accessible treatments for people with unmet medical needs. To achieve this goal, not only technological breakthroughs are required, but also the establishment of sustainable business models, talent cultivation systems, and medical reimbursement guarantee mechanisms, in order to integrate cell therapy into the routine clinical diagnosis and treatment process (Aboul-Soud et al., 2021). These practical challenges are extremely difficult and require in-depth participation of professionals from economics, public health, and health policy fields. For a long time, in the field of iPSC research, the relevant research forces in these disciplines have been relatively scarce, which is also an important factor limiting its rapid transformation.

## 6 Discussion and conclusions

The iterative development of iPSC reprogramming technology has enabled each new generation of techniques to overcome the limitations of the previous ones, while simultaneously revealing new challenges. From integrated viral vectors to non-integrated gene delivery systems, and from transcription factor induction schemes to chemical reprogramming techniques, it has consistently followed the development line from prioritizing efficiency to achieving safety and controllability, and from random uncertainty to precise intelligence (Guan et al., 2022; Peng et al., 2025; Wang Y. et al., 2025). This shift in the evaluation criteria from “technical feasibility” to “feasibility for large-scale production” marks the maturity of this research field.

The concept of “translational triltrilas” proposed in this article summarizes the inherent contradiction among safety, cellular homogeneity, and large-scale production, providing a clear analytical framework for explaining the lag in the development progress of this field. Although non-integrative delivery and chemical induction methods have significantly reduced safety risks, there are still potential safety hazards such as epigenetic abnormalities and genomic residual instability (Liuyang et al., 2023). Even though many breakthroughs have been made in single-cell characterization and epigenetic editing technologies, the problem of cellular homogeneity remains difficult to overcome. The individual differences of donor cells and the randomness of the reprogramming process continuously cause heterogeneity in cloned cells (Morris, 2025). Large-scale production, which has received the least attention from the academic community, is also the most difficult bottleneck to break through at present; this research direction needs to integrate bio-material engineering, microfluidic technology, automated equipment, and artificial intelligence technology to build a production platform, and the related supporting systems are currently still at the preliminary conception stage (Haase et al., 2025).

The breakthrough in future research will not come from the independent application of a single technology, but from the integration of technology and engineering platforms to achieve systematic parameter optimization and real-time quality prediction. Smart biomaterials can sense and respond to the state of cells, which is expected to build a closed-loop microenvironment and dynamically adapt to the changing physiological needs of reprogrammed cells (Indana et al., 2021). Microfluidic platforms are indispensable tools for high-throughput screening and process optimization due to their high precision and miniaturization (Jain S. et al., 2024). Artificial intelligence can sort out the complex parameter space and predict the cell quality based on early process data, which is expected to transform the empirically explored reprogramming technology into a predictable standardized scientific system (Nath et al., 2023). For industry practitioners, the above fusion scheme is the most feasible path to achieve low-cost, GMP-grade iPSC production, and also the core support to promote iPSC technology to clinical application.

Looking into the future, there are four core research directions that need to be focused on. First, the mechanism of chemical reprogramming needs to be further explored, especially the screening of small molecule targets and the elucidation of the intermediate state characteristics that can realize the epigenetic reprogramming of human cells (Chen et al., 2023; Liuyang et al., 2023). Second, it is necessary to optimize the combination scheme of CRISPR-based gene editing and reprogramming processes to improve the efficiency of gene editing under the premise of ensuring cell quality and genome integrity (Suter et al., 2025). Third, evaluation indicators that can predict cell quality are constructed based on single-cell multi-omics technology and machine learning algorithms, so as to realize real-time regulation of the experimental process and reduce the dependence on *post hoc* detection and analysis (Jain N. et al., 2024). Fourth, we should promote the synchronous development of the regulatory system, industrial economic support system and technology supporting iPSC therapy to ensure that safe and effective therapeutic products can benefit patients in a timely and affordable manner (Aboul-Soud et al., 2021).

Ultimately, the actual value of iPSC technology will depend not on the ingenuity of individual studies but on the sophistication of the systems that translate those discoveries into clinical applications. The field has already crossed the proof-of-concept stage, and is now facing the arduous task of building a complete system that integrates basic science, engineering technology and industry supervision to support the application of cell therapy. With the continuous integration of reprogramming technology, artificial intelligence-driven process regulation, and large-scale production platforms, the vision of iPSC cell therapy that was far away is gradually moving toward the clinic, and related research in the next decade is expected to truly realize this goal.
