# Supplementary material for: Induced pluripotent stem cell reprogramming: methodological evolution and challenges in clinical translation
Source: Front Cell Dev Biol. 2026 Aug 21;14:1931336. doi: 10.3389/fcell.2026.1931336 (PMC13542370; doi:10.3389/fcell.2026.1931336)
Supplement: Supplementary file 1 [file Table1.docx]

Supplemental Table S1： Comparative Characteristics of Major Reprogramming Strategies

| **Feature**  **Types** | **Integrating Viral Vectors** | **Episomal Plasmids** | **Non-integrative non-viral vectors** | **Chemical Small Molecule Induction** | **Gene Editing-Assisted Reprogramming** | **mRNA Transduction** | **PTD Protein Delivery** | **Nanotechnology Delivery Systems** |
| --- | --- | --- | --- | --- | --- | --- | --- | --- |
| **Genetic Material** | DNA (integrates into genome)[21] | DNA (extrachromosomal replication)[23, 121] | RNA (expressed in cytoplasm)[30, 35, 122] | Small molecule compounds (non-genetic)[4, 67] | DNA, RNA, or Protein (e.g., CRISPRa components)[123, 124] | RNA (translated in cytoplasm)[88, 125] | Protein (non-genetic)[47, 126] | DNA, RNA, or Protein (depends on payload)[49, 51, 55] |
| **Genome Integration Capability** | High[21, 22] | None[23, 24, 127] | None[30] | None[4, 67] | None (via endogenous gene activation)[123, 124] | None[88, 125] | None[46] | None (if payload is non-integrative)[49, 55] |
| **Site of Action** | Nucleus (genome)[21] | Nucleus (extrachromosomal replication & transcription)[23, 25, 121] | Cytoplasm (RNA replication & translation) [30, 122] | Cytoplasm & Nucleus (acts on signaling pathways & epigenetic targets)[128] | Nucleus (targets endogenous gene promoters)[123, 124] | Cytoplasm (mRNA translation)[125] | Cytoplasm & Nucleus (protein direct action)[47, 126] | Cytoplasm & Nucleus (depends on released substance)[49, 51] |
| **Possibility of Exogenous Gene Excision** | Difficult, requires gene editing [21] | Can be naturally lost during cell division or cleared via suicide gene systems[24, 127] | Can be naturally cleared after factor expression[122] | Unnecessary (metabolically degraded)[128] | Unnecessary (transiently activates endogenous program)[96, 124] | mRNA naturally degrades, no residue[40, 125] | Protein naturally degrades, no residue[47, 126] | Naturally degrades (if payload is non-integrative)[49, 51] |
| **Reprogramming Time** | 2-4 weeks [21] | 3-5 weeks [25] | 2-4 weeks [30] | 4-8 weeks[4, 67] | 3-6 weeks[124] | 2-4 weeks [40, 125] | 3-6 weeks[47, 126, 129] | 2-5 weeks[49, 51] |
| **Reprogramming Efficiency** | High[21, 22] | Low to Medium[23, 121] | Medium to High[30, 35] | Low[4, 67] | Medium to Low [124] | Medium to High[40, 125] | Low to Medium[129] | Potentially high but still needs optimization[49, 51] |
| **Technical Maturity** | Mature, early main method[21] | Relatively mature, especially episomal plasmids[121, 127] | Relatively mature, widely used in preclinical research[30, 35, 122] | Under development, successful paradigms exist[4, 61, 67] | Rapidly developing, shows great potential[96, 124, 130] | Rapidly developing, translated in other fields [125] | Under development, faces challenges like stability[46, 126] | Rapidly developing, various nanoparticles under R&D & optimization[49, 55] |
| **Principle** | Uses retrovirus or lentivirus to stably integrate reprogramming genes into host genome [21, 22] | Episomal plasmids replicate as independent forms outside nucleus, do not integrate; plasmid DNA is transiently expressed[24, 121, 127] | Viral vectors enter cells but do not integrate their genetic material; expressed then gradually cleared[30, 35, 122] | Uses specific small molecule compounds to induce cell reprogramming[128] | Targets endogenous pluripotency gene promoters via CRISPR activation (CRISPRa) system to induce expression[124] | Delivers encoding mRNA directly into cells, translated into protein in cytoplasm[125] | Delivers reprogramming factor proteins directly into cells[47, 126, 129] | Uses nanomaterials (e.g., polymers, lipid nanoparticles) to encapsulate & deliver reprogramming factors or genes[49-51, 55] |
| **Advantages** | High reprogramming efficiency, stable and long-lasting expression [21, 22] | No integration risk, relatively simple operation[24, 121, 127] | No genomic integration risk, higher safety [30, 35, 122] | Highest safety, no genetic material introduction[128] | Precisely activates endogenous genes, avoids exogenous gene introduction [96, 123, 124] | No integration risk, transient controllable expression[125] | Extremely high safety, no genetic material introduction[47, 126] | Strong designability, enables multi-functional co-delivery[49-51] |
| **Limitations** | Insertional mutations & tumorigenic risk, exogenous genes may interfere with differentiation[21, 22] | Relatively low efficiency, plasmids may be lost[24, 121, 127] | May have low efficiency, viral residue or immune response[30, 122] | Low efficiency, long cycle, complex mechanisms [128] | Efficiency needs to be improved, and there is an off-target risk[96, 123, 124] | mRNA easily degraded, requires multiple transfections, high cost[40, 125] | Protein instability, low efficiency, high production cost[47, 126] | Delivery efficiency and biocompatibility require optimization, and the challenges of in vivo applications are significant [49-51] |
| **Clinical Application Potential** | Limited due to insertional mutation risk[21] | Relatively high clinical potential, applied in preclinical research[28, 127] | Relatively high clinical potential, used in preclinical research[122] | High potential, expected to achieve footprint-free reprogramming [61, 128] | High clinical potential, enables precise endogenous gene regulation[124] | High clinical potential, validated in other therapies[40, 125] | Medium potential, requires solving efficiency & stability[47, 126] | High potential, enables targeted & smart delivery[49, 55] |
| **Application Mode** | Mainly for basic research, exploring reprogramming mechanisms[21] | Widely used in preclinical research, disease modeling, drug screening, and is expected to be used in clinical practice [28, 127] | Widely used in preclinical research, disease modeling, drug screening[30, 122] | Drug screening, optimizing reprogramming, potential for footprint-free reprogramming [61, 128] | Basic research, gene function studies, cell fate regulation, iPSC reprogramming[130, 131] | Widely used in CAR-T cell therapy, vaccine development, gene therapy, iPSC reprogramming[40, 125] | Basic research, proof-of-concept[46, 126] | Gene therapy, drug delivery, iPSC reprogramming, future clinical applications [49, 55] |
